# Maternal Anemia as a Predictor of Childhood Anemia: Evidence from Gambian Health Data

**DOI:** 10.3390/nu17050879

**Published:** 2025-02-28

**Authors:** Alhagie Sowe, Elizabeth Wood, Santosh Kumar Gautam

**Affiliations:** 1 Eck Institute for Global Health, University of Notre Dame, Notre Dame, IN 46556, USA; 2 Keough School of Global Affairs, University of Notre Dame, Notre Dame, IN 46556, USA

**Keywords:** iron deficiency anemia, maternal anemia, children, rural, socioeconomic status, The Gambia

## Abstract

Background: Iron deficiency anemia (IDA) is a significant global health problem affecting close to 2 billion people worldwide. The prevalence of IDA is higher among children younger than five years and women of reproductive age, indicating an intergenerational correlation between maternal and child anemia. This study aims to analyze the association between maternal and child anemia in The Gambia. Methods: A nationally representative dataset comprising 3249 children under the age of five, obtained from The Gambia Demographic and Health Survey (2019–2020), was utilized for empirical analyses. Multivariate linear regression models were employed to assess the association between maternal and child anemia. The multivariate models were adjusted for various confounding variables, including birth order, age, and the gender of the child, as well as maternal education, religion, wealth quintiles, rural residence, and region-fixed effects. Results: Fifty-three percent of children and 52% of mothers are anemic. Children from poorer households show a higher rate of anemia compared to those from wealthier households. Maternal anemia was significantly associated with the anemia status of the children. Children born to anemic mothers were 13.5% more likely to be also anemic (*p* < 0.001). The correlation coefficient between mother and child hemoglobin levels is 0.165 (*p* < 0.001). The correlation coefficient between maternal and child anemia is higher among the bottom three wealth quintiles than the top two wealth quintiles. Conclusions: The significant intergenerational association between maternal and child anemia status highlights the need for targeted, multi-pronged strategies to combat the adverse impacts of anemia. Maternal anemia, in general, appears to influence childhood anemia beyond just the pregnancy period. Shared socioeconomic environment, dietary patterns, and exposure to infections likely contribute to this intergenerational association.

## 1. Introduction

Anemia is a condition in which the number of red blood cells or the concentration of hemoglobin within them is below normal levels, impairing the blood’s ability to carry oxygen efficiently [1]. It is a major global health issue, particularly affecting children younger than five, pregnant and postpartum women, and menstruating adolescent girls and adult women [1]. Among the various types of anemia, the most prevalent is iron deficiency anemia (IDA), a condition closely linked to increased maternal mortality risk [2].

Iron deficiency occurs when the body’s iron stores are depleted. This can progress to IDA, characterized by low iron levels, anemia, and the presence of microcytic hypochromic red blood cells—smaller, paler cells that are indicative of the anemic condition [3]. IDA not only reflects a poor nutritional and health status but also poses serious risks, particularly for young children. Severe anemic conditions in children may result in impaired growth, developmental delays, and cognitive disabilities [4]. In adults, IDA significantly affects physical performance and work productivity, leading to broader health and economic repercussions [5,6]. Addressing IDA through interventions such as iron supplementation has proven to be a highly cost-effective strategy, offering substantial improvements in worker productivity and overall quality of life [7].

A global burden of disease study found that, although the global prevalence of IDA across all ages declined from 28.2% in 1990 to 24.3% in 2021, the total number of individuals affected by anemia increased from 1.5 billion to 1.92 billion during the same period [8]. This study further found considerable differences in prevalence across various regions, sexes, and age groups [8]. Geographically, the regions with the highest anemia prevalence were western sub-Saharan Africa (47.4%), South Asia (43.0%), and central sub-Saharan Africa (35.7%), both in the 1990 data and in the most recent 2021 data. Children under five years of age and women living in sub-Saharan Africa (SSA) and South Asia bear the highest burden of anemia [8]. In SSA, the anemia rate among children aged 6–23 months is alarmingly high, affecting 76.6% of this group [9]. The prevalence of anemia in SSA shows significant variation across severity levels and age groups. Among children aged 6–59 months, 64.1% experience anemia. This breaks down to 26.2% with mild anemia, 34.9% with moderate anemia, and 3% with severe anemia [10]. Analysis of 27 countries in SSA revealed that 43.3% of married women in this region were anemic, with particularly high rates being seen in central and western Africa [11]. Factors associated with a higher risk of anemia among children and women in SSA include poverty, low maternal education, infectious diseases such as malaria and HIV, undernutrition, and limited access to healthcare [9,10,11,12].

In addition to iron deficiency, megaloblastic anemia caused by vitamin B12 and folate deficiencies is another important nutritional cause of anemia in developing countries. These deficiencies often coexist with iron deficiency in malnourished populations, contributing to the overall burden of anemia. Megaloblastic anemia affects cellular development and can lead to cognitive impairment, developmental delays, and other adverse health outcomes [13,14]. In pregnant women, folate deficiency specifically increases the risk of neural tube defects and other pregnancy complications [15].

In addition to household socioeconomic status and exposure to infectious disease, several studies have identified maternal anemia as a key predictor of childhood anemia primarily due to shared nutritional deficiencies, genetic predispositions, dietary habits, and environmental exposures [10,16]. Furthermore, most research has focused on broad regional trends rather than specific country-level analyses that account for local socioeconomic and healthcare disparities. This study addresses this gap by examining the intergenerational association between maternal and child anemia using nationally representative data from The Gambia, a small country in West Africa. We also investigate whether the intergenerational association varies based on the sociodemographic characteristics of the mothers, such as the mother’s education, the age of the child, and household wealth quintiles.

According to the 2019–2020 Gambian Demographic Health Survey (GDHS), the prevalence of anemia among pregnant women was 44% [17], a decrease from the 2013 GDHS, which reported 68% [18]. In nationally representative data on 0–59-months-old children, the prevalence of anemia was estimated to be around 50.4%, and nearly 40% of anemia was due to iron deficiency, according to The Gambia Micronutrient Survey [19]. Significant predictors of anemia prevalence among children younger than five in the Gambia included maternal anemia level, the birth order of children, children’s age in months, wasting, underweight, stunting, household wealth status, history of breastfeeding, sex of the household head, and ethnicity [20]. The odds of anemia occurring among children who are from anemic mothers is 57.7% (AOR = 1.577; 95% CI: 1.308–1.902) higher than those from non-anemic mothers [20]. Other contributing factors include vitamin A deficiency, infectious diseases (such as malaria), and nutritional status. Factors that increase the risk of higher levels of anemia among Gambian children include poor maternal education, lower household wealth, larger family sizes, being male, multiple births, recent fever or diarrhea, higher birth order, maternal anemia, and undernutrition (underweight, wasting, stunting) [10].

Conversely, factors that reduce the risk of anemia include being 24–59 months of age, taking drugs for intestinal parasites, and being born to mothers aged 20 years or older [10]. The prevalence of anemia in children aged 6–59 months has declined from 73% in 2013 to 45% in 2019–2020, according to the GDHS report [17]. The reduction in anemia rates among children is strongly correlated with factors such as increased maternal education and improved household wealth [10,16,19,20]. Despite this overall progress, significant regional disparities persist in anemia prevalence across different parts of the country. For instance, children in rural areas exhibit a markedly higher rate of anemia (60%) compared to those in urban areas (37%). Disparities are also evident within smaller geographic regions. Anemia prevalence varies widely across local government areas, with a range from 30% in Brikama to 77% in Kuntaur [17]. These findings suggest that, while broader socioeconomic improvements contribute to lower anemia rates, targeted interventions are necessary to address these regional disparities.

## 2. Methods

### 2.1. Study and Setting

The Gambia is a small West African country with a population of 1.88 million, nearly half of which is rural. It is one of the most densely populated countries in sub-Saharan Africa, with a population growth rate of 3.1% per year, dominated by youth [21]. The Gambia is classified as a low-income country, with a GDP per capita of USD 808.28 in 2022 and more than half of the population living below the international poverty line of USD 1.25 per day [22]. Poverty is significantly higher in rural areas, coupled with widespread food insecurity. The rural poor relied heavily on agriculture for their livelihoods, which accounts for 70% of the total labor force of the country [22,23].

However, the agriculture sector is characterized by subsistence farming, low productivity, declining soil fertility, and reliance on rain-fed cultivation [23]. The prevalence of food insecurity was 29% in 2023, an increase of 3% from the previous year. Food insecurity is notably more prevalent in rural areas, affecting 52% of the population, whereas urban regions experience a significantly lower rate of 21% [24]. The cost of living has soared since 2021, largely due to disruptions in the global supply chain caused by the COVID-19 pandemic and the Russia–Ukraine crisis. These disruptions have driven food prices up nationwide [23,24].

### 2.2. Patient

Patients and the public were not involved in the design and conducting of this research.

### 2.3. Data

This study utilizes the 2019–2020 GDHS, a comprehensive, nationally representative household survey conducted between 21 November 2019 and 30 March 2020. The survey included a sample of 6549 households and 11,865 women aged 15–49. The GDHS gathered representative data on various indicators including sociodemographic factors, fertility, family planning, maternal and child health, and nutrition.

For anemia assessment, blood samples were collected from consenting women aged 15–49 and children aged 6–59 months. These samples were obtained via microcuvette and analyzed on-site using the HemoCue 201+ device to determine hemoglobin levels. The HemoCue is a point-of-care testing device that measures hemoglobin levels in blood. The HemoCue machine is manufactured by a Sweden-based medical equipment company called the HemoCue Ab. The HemoCue machine was procured by the research team from its manufacturer in India. The GDHS data collected hemoglobin (Hb) data from all children aged 6–59 months in half of the households eligible for Hb testing, and Hb testing was conducted for 95% of eligible children, yielding a sample of 3249 children [17]. Results were shared with the participants both verbally and in writing.

### 2.4. Variables

The primary outcome variables are Hb levels and the anemic status of 6–59-month-old children. Both outcomes were measured at the time of the survey. Hb levels are continuous variables reported in grams per decilitre (g/dL). Anemic status is a dichotomous variable, with a value of “1” indicating anemic status and “0” indicating non-anemic status. The World Health Organization (WHO) defines anemia as Hb levels <11.0 g/dL in children younger than five and pregnant women and <12.0 g/dL in non-pregnant women [1]. The primary exposure variables are either the mother’s Hb levels (continuous) or “maternal anemia”—a binary variable indicating the anemic status of the mothers based on the WHO classification. The gender of the child, religion, and rural residence are included in the analysis as binary variables, whereas birth order, age of the child, and maternal education are included as continuous variables. The household wealth index is constructed using data on asset ownership and housing characteristics. Assets include consumer goods (such as televisions, bicycles, or cars), while housing characteristics encompass drinking water sources, toilet facilities, and flooring materials. Using principal component analysis, these variables are combined to generate a composite wealth score for each household. This score is then assigned to all usual (permanent) household members. The population is ranked according to these individual scores and divided into five equal groups (quintiles), with each quintile containing 20% of the population. This methodology creates a relative measure of household socioeconomic status based on assets rather than income or consumption [17].

Per the 2019-20 GDHS report, 44% of women had some form of anemia; 26% were mildly anemic, and 17% were moderately anemic [17]. Pregnant women were more likely to be anemic (55%) than women who were breastfeeding (47%) and women who were neither breastfeeding nor pregnant (42%). Maternal anemia was higher in rural areas (56%) than in urban areas (40%). The prevalence of anemia was lowest in Brikama (39%) and highest in Kuntaur (62%) [17]. We chose to use a complete-case analysis approach, excluding observations with missing values rather than employing statistical imputation techniques. This approach yielded a final analytical sample of 3249 children with complete hemoglobin, anemia information for mother-child pairs, and confounding variables.

### 2.5. Statistical Analysis

This study employed a multivariate linear regression model to examine the association between maternal and child anemia. When trying to obtain Hb levels as the outcome, a linear regression model is appropriate for the analysis. For the binary outcome of anemia, the study uses a Linear Probability Model (LPM). An LPM is an application of a linear regression model to binary outcomes. While we prefer an LPM over non-linear models, such as logistic/probit models, because the estimated parameters are easy to interpret, we acknowledge its potential limitations when analyzing binary outcomes like anemia status. Unlike logistic regression, an LPM can predict probabilities outside the [0, 1] interval and assumes constant marginal effects. However, as shown by Hellevik (2007), LPM coefficients often closely approximate the marginal effects from logistic regression, particularly when probabilities are not close to 0 or 1, as is the case in our study where anemia prevalence is around 50% [25]. We conducted robustness checks using logistic regression and found similar results, suggesting our choice of LPM does not substantially affect our conclusions. We estimate the following regression models:

(1) $$Y_{cmr}=\alpha +\beta_{1}M_{mr}+\beta_{2}X_{m}+\mu_{r}+\epsilon_{cmr}$$

where $\;Y_{cmr}$ is either the Hb level or anemic status of child *c* born to mother *m* in region *r*. $M_{mr}\;$ is the Hb levels/anemic status of mothers *m* in region *r*. $X_{m}$ are the confounding variables that may impact the anemic status of children. These confounding variables include child-level variables such as gender, birth order, and age of the children; mothers’ characteristics such as maternal education; household characteristics including religion, wealth quintiles, and residence (rural versus urban). $\mu_{r}$ is the region’s fixed effects to account for fixed characteristics of the regions. Robust standard errors are estimated in all regression models. Data analysis was carried out using STATA version 18. We also conducted subgroup analyses to examine whether the correlation between maternal and child anemia differs by the mother’s characteristics. We estimate the regression model outlined in Equation (1) separately by the mother’s education, the age of the child, and household wealth quintiles.

## 3. Results

**Figure 1:**
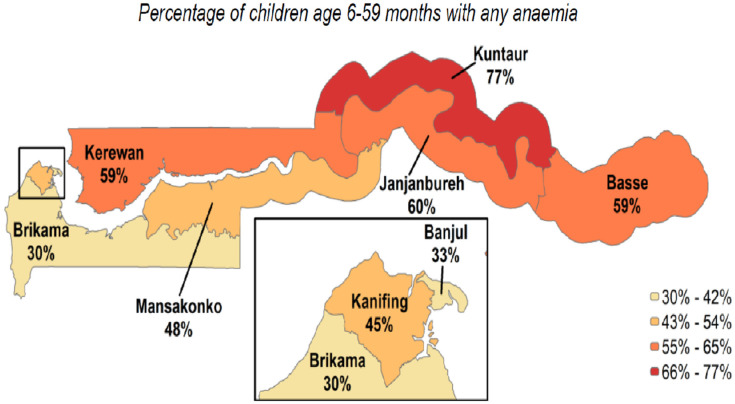
Anemia among children aged 6–59 months, by the local government area. Source: Gambian Demographic Health Survey, 2019.

First, we show the prevalence of anemia across different regions. We report these summary descriptions from the GDHS data. It should be noted that the geographical variation across regions in Figure 1 is not based on our analytical sample but is from the GDHS report [17]. Approximately 45% of under-five children were anemic (Hb level below 11.0 g/dL). Of children suffering from any form of anemia, 24% had mild anemia (Hb 10.0–10.9), 20% had moderate anemia (7.0–9.9 g/dL), and only 1% had severe anemia (<7.0 g/dL). The Hb levels are adjusted for altitudes that are above 1000 m. The GDHS shows that the prevalence of anemia is higher among children in rural areas (60%) than in urban areas (37%). The percentage of anemic children ranges from 30% in the Brikama region to 77% in the Kuntaur region (Figure 1).

### 3.1. Population Description

Table 1 presents the descriptive statistics of the analytical data. It reveals that over half of the children and mothers in the study sample are anemic, with 53% of children and 52% of mothers showing signs of any form of anemia. The mean birth order is four children per mother, with 48% of the children being female. Additionally, over 50% of households are categorized as living in poverty (bottom two quintiles), and 39% of the sample population reside in rural areas. The majority of the sample is Muslim (98%), and the average maternal years of schooling is 3.57 years. These statistics suggest significant socioeconomic and health disparities within the sample group.

### 3.2. Multivariate Regression Results

Table 2 displays the intergenerational correlation in nutritional outcomes between mothers and their children. Columns 1–2 show the correlation for Hb levels, and columns 3–4 show the correlation for anemia. After adjusting for child-specific and household-specific controls, along with regional fixed effects, it is observed that a mother’s Hb levels are significantly associated with the Hb levels of her children. The Hb correlation coefficient of 0.165 implies that if mothers’ Hb levels increase by 1 g/dL, the Hb levels of children increase by 0.16 g/dL. Furthermore, results in column 4 show that, when a mother is anemic, the likelihood of her children also becoming anemic increases by 13.5%. These intergenerational coefficients are statistically significant at a 1% level of significance. These results highlight a significant intergenerational association in anemia, emphasizing the importance of addressing maternal anemia to reduce the risk of anemia in children.

We also conducted a robustness check using a logistic regression model for the anemia outcome. The results in Table S1 show a strong and statistically significant association between maternal and child anemia. Children born to anemic mothers had 89% higher odds of being anemic compared to children of non-anemic mothers (OR = 1.89, 95% CI: 1.62–2.21, *p* < 0.001). This substantial increase in odds persisted even after controlling for other maternal, child, and household characteristics. Maternal education also showed a significant, though more modest, relationship with child anemia. These logistic regression results are qualitatively similar to the LPM results in Table 2, suggesting that our choice of linear model does not substantially affect our main conclusions.

In Table 3, we explore whether the association between maternal and children anemia reported in Table 2 varies by the age of the children and maternal education. Since dietary patterns are different for younger children than older children, it is hypothesized that the intergenerational association in anemia may be different for younger versus older children. We analyzed the sample for children aged less than 12 months and more than 12 months. We found that intergenerational correlation is higher (0.16) for children less than 12 months old compared with children more than 12 months old (0.13). When we analyzed the data by mother’s education, the coefficients were similar between mothers who have completed primary school and mothers who did not complete primary school (0.138 versus 0.129). All coefficients reported in Table 3 are statistically significant at a 1% level of significance.

Our findings in Table 2 and Table 3 show that maternal anemia increases child anemia risks significantly, suggesting that every prevented case of maternal anemia could potentially protect multiple children from developing anemia. This relationship could support several specific intervention approaches: (1) expanding preconception care and iron supplementation for women of reproductive age, not just during pregnancy; (2) implementing maternal anemia screening as an early warning system to identify children at high risk; and (3) designing family-based interventions that address both maternal and child anemia simultaneously through combined supplementation and dietary improvement programs.

**Figure 2:**
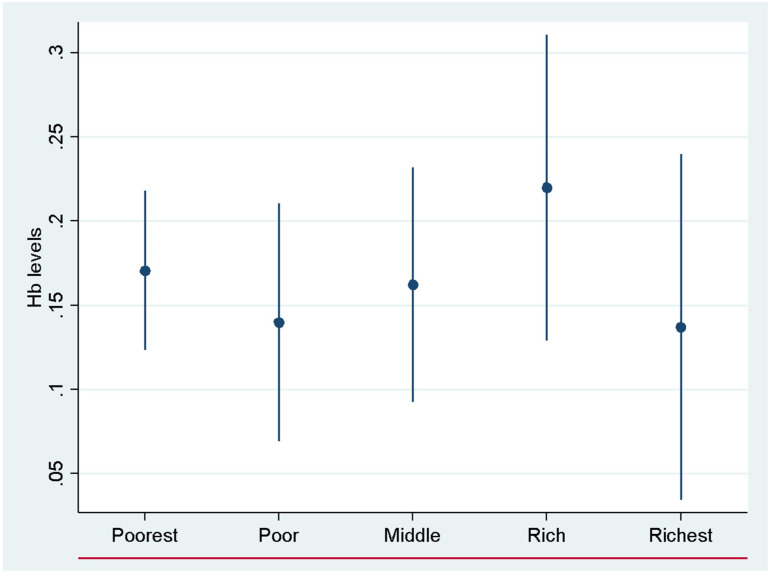
Heterogeneous effects on hemoglobin by wealth quintiles. Each dot is a regression coefficient estimated separately for each group. The bar shows a 95% confidence interval. The red line shows Hb equals 0.

**Figure 3:**
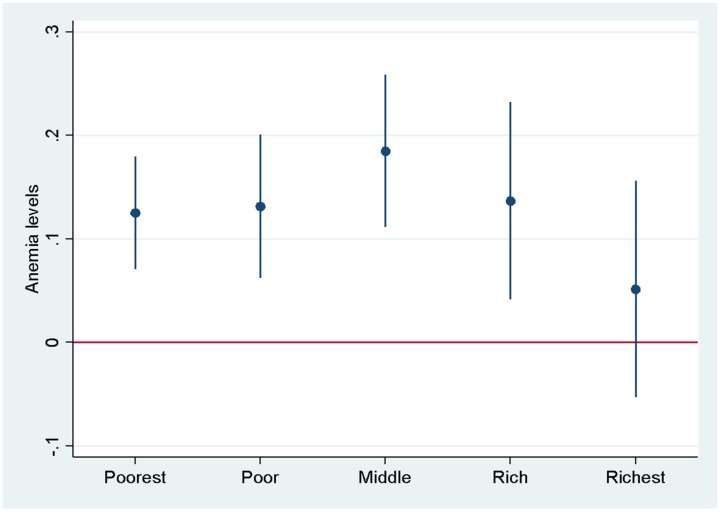
Heterogeneous effects on anemia by wealth quintiles. Each dot is a regression coefficient estimated separately for each group. The bar shows a 95% confidence interval.

Finally, we analyzed these associations by household wealth quintiles. The results of this study demonstrate a significant intergenerational association between maternal and child anemia status among households in the three lowest wealth quintiles compared to those in the top two quintiles (Figure 2 and Figure 3). The bar line in Figure 2 and Figure 3 shows the 95% confidence interval. Children born to mothers in the poorest households (bottom three wealth quintiles) had a substantially higher risk of developing anemia compared to children born in the richest wealth quintiles (Figure 3). Results by wealth quintiles show a clear socioeconomic disparity in the mother–child transmission of anemia. These findings suggest that children from poorer households face a significantly higher risk of anemia, likely due to factors such as poor maternal nutrition, limited access to healthcare, and suboptimal infant and young child feeding practices.

The inverted U-shape relationship shown in Figure 3, where the maternal–child anemia association is strongest among middle and lower wealth quintiles but weaker in the highest quintiles and has relevance for targeted public health interventions based on socioeconomic characteristics of the households. This pattern suggests that wealthier households may have protective factors—such as better access to healthcare, diverse nutrition, and sanitation facilities—that help break the intergenerational transmission of anemia. Addressing these disparities could involve expanding maternal and child nutrition programs, improving access to iron supplementation, and enhancing healthcare delivery in resource-constrained areas. These findings indicate that integrated interventions addressing both direct nutrition support and underlying socioeconomic barriers may be more effective than isolated nutritional interventions. Targeted programs for middle- and lower-income households might include combining iron supplementation with improved access to health services, nutrition education, anemia screening, and economic support for vulnerable populations.

## 4. Discussion

More than half of the children (53%) and mothers (52%) are anemic in The Gambia. After adjusting for various individual and community-level factors, we find that maternal anemia was significantly associated with childhood anemia, which is consistent with findings in previous studies [26,27,28,29,30]. These findings underscore the importance of maternal health status, particularly maternal anemia, as a key predictor of child anemia outcomes.

Our findings are consistent with a prior study where maternal anemia was a significant predictor of anemia among children younger than five within a Sudanese population [26]. Ntenda and colleagues (2018) also found that maternal anemia was strongly associated with childhood anemia in four southern African countries (Malawi, Mozambique, Namibia, and Zimbabwe), revealing that maternal anemia and communities with a high percentage of anemic women have adverse effects on childhood anemia extending beyond pregnancy [27]. This can be explained by the fact that mothers with anemia often live in poor households or communities, indicating socioeconomic deprivation. This can lead to inadequate intake of iron and other essential nutrients, resulting in anemia for both mothers and their children. Since they share similar social, environmental, and household factors after birth, their dietary patterns and quality of life are often alike. They are also exposed to similar infections, such as intestinal parasites and malaria, which can impact red blood cell production and iron stores. Additionally, low levels of essential minerals and vitamins in the breast milk of anemic mothers can further affect the hemoglobin levels of breastfeeding children [28].

Similarly to our findings, studies across African countries have reported a higher risk of anemia among younger children, with the cause likely being a combination of demand based on the growth rate of the infant and the potential risk of disease (e.g., diarrhea) as the infant is weaned off of breastmilk [28,29,31]. Anemia, large family sizes, and poverty are often interconnected factors that contribute to poor maternal and child health outcomes. This high prevalence of anemia in low socioeconomic households in The Gambia and sub-Saharan Africa in general has been well documented [17,32,33].

The results of this study demonstrate a significant intergenerational association in anemia status. Maternal anemia appears to be an important risk factor for anemia in children, even after accounting for other potential influences. If a mother is anemic, the likelihood of her children also being anemic increases by 13.5%. The correlation coefficient between maternal and child Hb levels is 0.16, indicating a modest positive relationship. This complex relationship is a result of various intrinsic and extrinsic factors, including socioeconomic status, maternal nutrition, wealth index, place of residence, and access to healthcare facilities, as previously reported [32,33,34,35].

Overall, the high prevalence of anemia in the study population represents a serious concern from an economic standpoint. Our findings show that children from the poorest households have significantly higher odds of developing anemia, with the strongest intergenerational transmission of anemia occurring in the bottom three wealth quintiles. This implies that anemic adults, particularly women, may experience reduced work capacity and productivity, which may limit their earning potential and ability to provide adequate nutrition for their families. Several studies have found that anemia negatively impacts occupational performance and productivity [13,36,37]. In The Gambia, where 70% of the labor force relies on agriculture for their livelihood, the physical demands of farming make this productivity loss particularly devastating. Prior studies show that median annual productivity losses due to iron deficiency anemia can account for approximately 0.57% of a country’s GDP [37]. Breaking this intergenerational cycle of iron deficiency through targeted anemia reduction programs could yield substantial economic benefits. By improving maternal health and work capacity, such interventions could enhance household income, agricultural productivity, and children’s nutritional status. Given that over half of The Gambia’s population lives below the international poverty line of USD 1.25 per day, investments in anemia prevention could serve as a crucial pathway for poverty reduction, particularly in rural areas where both anemia prevalence and poverty rates are highest. Furthermore, anemia can affect poverty through diminished human capital formation among school-age children. Anemia is associated with reduced oxygen delivery to tissues, leading to fatigue and decreased physical activity levels, and lower cognitive function and attention spans, which, in turn, could affect the educational outcomes of children [8,38]. The negative consequences of anemia on education may prevent vulnerable populations from overcoming the intergenerational poverty traps.

Given its multifaceted causes, tackling childhood anemia requires a comprehensive, multi-sectoral approach integrating maternal and child nutrition programs, infection control measures, strengthened healthcare systems, and socioeconomic interventions [2]. Fortifying staple foods [31], expanding access to iron and folic acid supplementation [39], use of double fortified salt [40,41], integrating malaria and deworming programs [10], improving antenatal care [17], and promoting dietary education [42] are all critical strategies to address anemia burden in SSA. Additionally, poverty reduction efforts and food security programs must be prioritized to ensure sustainable improvements in child health [21]. A coordinated public health response that addresses both biological and social determinants is essential to breaking the cycle of anemia and improving long-term health outcomes for children in Africa [31,33].

## 5. Limitations of the Study

This study utilizes primary data from the GDHS. The GDHS is cross-sectional and does not account for seasonal changes in anemia status for both mothers and children. This can affect the actual estimates of anemia at the population level. The data are also vulnerable to recording and recall bias from the side of the field workers and participants, respectively. Furthermore, the results described in this study are correlational and do not establish a cause–effect relationship between maternal and child anemia. Therefore, our findings should not be interpreted as the causal association between maternal and child anemia. Another issue could be related to sample selection bias due to survivor bias. The DHS data includes children who survived until the data collection, and severely anemic children may likely have not survived and, thus, were missed from the DHS sample. Other factors such as malaria and other intestinal parasitic infections and dietary patterns affect anemia prevalence; however, due to the unavailability of data on these factors, the current study was unable to include them in the regression analysis. Furthermore, due to data limitations in the GDHS, which primarily measures hemoglobin levels without differentiating between types of anemia such as megaloblastic anemia, our analysis focuses on overall anemia status with particular attention to iron deficiency anemia as the predominant form in our study population.

## 6. Conclusions

The high prevalence of anemia, socioeconomic disparities, and the intergenerational association between maternal and child anemia status highlight the need for targeted, multi-pronged strategies to combat this public health challenge. Maternal anemia, in general, appears to have an influence on childhood anemia beyond just the pregnancy period. Shared socioeconomic environment, dietary patterns, and exposure to infections likely contribute to this intergenerational association. Additionally, genetic predispositions, such as inherited variations in genes controlling iron absorption, transport, and metabolism, can affect susceptibility to anemia across generations. For instance, genetic variants affecting iron regulatory proteins or hemoglobin synthesis could make certain families more vulnerable to developing anemia even under similar environmental conditions. Furthermore, disparities in healthcare access can perpetuate anemia transmission across generations—mothers with limited healthcare access during pregnancy may be more likely to have anemic children, who, in turn, face the same structural barriers to treatment. The combined effect of genetic predisposition and healthcare access inequities may help explain why the intergenerational correlation in anemia is particularly strong among lower wealth quintiles, as shown in our analysis.

The strong intergenerational association suggests that interventions targeting maternal anemia could have significant benefits for child health outcomes. Addressing anemia in women, especially during pregnancy and postpartum, is crucial to breaking the cycle of anemia and improving child health outcomes. Interventions should target both maternal and child nutrition. School-based interventions, such as the expansion of the iron-fortified school lunch program [40,41] and the distribution of iron supplements every week, could help tackle anemia among school children. Iron supplementation for pregnant women and children, nutrition education sessions and counseling, and monthly anemia screening services at the community level may help address anemia among women. Economic support to provide subsidies for iron supplements and the purchase of fortified food products could also be implemented to improve maternal nutrition among poor families in rural areas.

Anemia has serious consequences for health, development, and productivity. Failure to reduce anemia can impair cognitive and motor development in children and work capacity in adults and lead to adverse pregnancy outcomes. If left unaddressed, childhood anemia can perpetuate a cycle of poor educational attainment, reduced economic productivity, and an increased burden on already stressed healthcare systems, ultimately jeopardizing the development of entire communities. The widespread impact of childhood anemia underscores the importance of implementing public health policies that ensure early detection, adequate nutrition, and access to quality healthcare.

## Figures and Tables

**Table 1:** Descriptive statistics of sample data (N = 3249).

Variables	Mean	Std Dev	Min Value	Max Value
Health Outcomes				
Child Hb	10.57	14.36	27	144
Mother Hb	11.64	15.26	33	185
Child is anemic (%)	0.53	0.5	0	1
Mother is anemic (%)	0.52	0.5	0	1
Child Demographics				
Birth order	3.71	2.34	1	10
Child age (months)	27.52	17.5	0	59
Female (%)	0.48	0.5	0	1
Household Demographics				
Mother’s education	3.57	4.46	0	20
Religion (Muslim)	0.98	0.11	0	1
Socioeconomic Status				
Poorest	0.35	0.48	0	1
Poor	0.22	0.41	0	1
Middle	0.19	0.39	0	1
Rich	0.13	0.34	0	1
Richest	0.1	0.3	0	1
Rural (%)	0.39	0.49	0	1
No. of local government regions	8			

Notes: Hemoglobin (Hb) levels are reported in g/dL.

**Table 2:** Association between maternal–child hemoglobin levels and anemia.

	Child’s Hb Level		Child Is Anemic	
	(1)	(2)	(3)	(4)
Mother’s Hb level	0.181 ***	0.165 ***		
	(0.0155)	(0.015)		
Mother is anemic			0.147 ***	0.135 ***
			(0.0167)	(0.016)
Child controls	Yes	Yes	Yes	Yes
Household controls	Yes	Yes	Yes	Yes
Region-fixed effects	No	Yes	No	Yes
Observations	3249	3249	3249	3249

Notes: Robust standard errors are reported. Coefficients from linear regression models are shown. All models are adjusted for age, gender, and birth order of the child; mother’s education, religion, rural residence, and wealth quintiles. *** indicates *p* < 0.001.

**Table 3:** Intergenerational correlation in Hb and anemia, by mother’s education and child’s age.

	Child Is Anemic			
	Children’s Age		Mother’s Education	
	<12 months	≥12 months	<5 years	≥5 years
	(1)	(2)	(3)	(4)
Mother is anemic	0.16 ***	0.13 ***	0.138 ***	0.129 ***
	(0.05)	(0.017)	(0.021)	(0.027)
Child controls	Yes	Yes	Yes	Yes
Household controls	Yes	Yes	Yes	Yes
Region-fixed effects	No	Yes	No	Yes
Observations	373	2874	1994	1255

Notes: Robust standard errors are reported. Coefficients from linear regression models are shown. All models are adjusted for age, gender, and birth order of the child, as well as the mother’s education, religion, rural residence, and wealth quintiles. *** indicates *p* < 0.001.

## Data Availability

The data that support the findings of this study are available and can be requested from the website of the DHS Program (https://dhsprogram.com/data (accessed on 20 January 2024)).

## Supplementary Materials

The following supporting information can be downloaded at: https://www.mdpi.com/article/10.3390/nu17050879/s1, Table S1: Logistic regression analysis of factors associated with anemia among children in The Gambia.
