# Supplementary material for: Maternal Anemia as a Predictor of Childhood Anemia: Evidence from Gambian Health Data
Source: Nutrients. 2025 Feb 28;17(5):879. doi: 10.3390/nu17050879 (PMC11901893; doi:10.3390/nu17050879)
Supplement: Supplementary file 1 [file nutrients-17-00879-s001.zip › nutrients-3495266-supplementary.pdf]

Table S1. Logistic regression analysis of factors associated with anemia among children in The Gambia

Variable	Odds Ratio	Robust Std. Err.	P-value	95% CI
Maternal Characteristics				
Maternal anemia	1.89	0.15	<0.001	1.62-2.21
Maternal education	0.91	0.04	0.049	0.83-1.0
Child Characteristics				
Female	0.81	0.06	0.005	0.69-0.94
Birth order	0.98	0.02	0.219	0.95-1.01
Child age (months)	0.97	0.003	<0.001	0.96-0.97
Household Characteristics				
Religion (Muslim)	0.33	0.15	0.017	0.14-0.82
Rural residence	1.01	0.08	0.901	0.86-1.19
Wealth Quintile (ref: poorest)				
Poor	0.69	0.07	0.001	0.56-0.85
Middle	0.63	0.08	<0.001	0.50-0.80
Rich	0.63	0.10	0.002	0.47-0.85
Richest	0.49	0.09	<0.001	0.35-0.69
Region (ref: Banjul)				
Kanifing	1.69	0.36	0.013	1.12-2.55
Brikama	0.66	0.13	0.040	0.45-0.98
Mansakonko	1.07	0.24	0.763	0.69-1.65
Kerewan	1.89	0.41	0.003	1.24-2.88
Kuntaur	3.59	0.82	<0.001	2.29-5.61
Janjanbureh	1.80	0.39	0.008	1.17-2.76
Basse	1.96	0.39	0.001	1.32-2.91
Constant	8.52	4.50	<0.001	3.03-24.0

Notes: N = 3,249; Pseudo R^2 = 0.1274; Wald $\chi^2(18) = 453.59$, $p < 0.001$ CI = Confidence Interval; ref = reference category. The model estimates the odds of a child being anemic. All standard errors are robust.
